# Femoral vessel cannulation strategies in minimally invasive cardiac surgery: single Perclose ProGlide versus open cut-down^†^

**DOI:** 10.1093/ejcts/ezaf118

**Published:** 2025-04-03

**Authors:** Majd Ismail, Dilara Merve Berberoglu Aydin, Jang-Sun Lee, Markus Schönburg, Efstratios Charitos, Yeong-Hoon Choi, Oliver Joannis Liakopoulos

**Affiliations:** Department of Cardiac Surgery, Kerckhoff Clinic Bad Nauheim, Campus University of Giessen, Bad Nauheim, Germany; Department of Cardiac Surgery, Kerckhoff Clinic Bad Nauheim, Campus University of Giessen, Bad Nauheim, Germany; Department of Cardiac Surgery, Kerckhoff Clinic Bad Nauheim, Campus University of Giessen, Bad Nauheim, Germany; Department of Cardiac Surgery, Kerckhoff Clinic Bad Nauheim, Campus University of Giessen, Bad Nauheim, Germany; Department of Cardiac Surgery, Kerckhoff Clinic Bad Nauheim, Campus University of Giessen, Bad Nauheim, Germany; Department of Cardiac Surgery, Kerckhoff Clinic Bad Nauheim, Campus University of Giessen, Bad Nauheim, Germany; Department of Cardiac Surgery, Kerckhoff Clinic Bad Nauheim, Campus University of Giessen, Bad Nauheim, Germany

**Keywords:** Minimal invasive surgery, Percutaneous vascular closure device, ProGlide, Vascular complication

## Abstract

**OBJECTIVES:**

Surgical cut-down is commonly used for femoral cannulation in minimally invasive cardiac surgery, but is associated with higher rates of wound healing disorders. Percutaneous cannulation using vascular closure devices offers a less invasive alternative with potentially fewer complications. The aim of this study was to assess the feasibility and safety of using a single ProGlide system for primary access-site closure in comparison with surgical cut-down. Additionally, we analysed the learning curve for adopting this strategy.

**METHODS:**

A retrospective analysis was conducted on 753 patients who underwent minimally invasive cardiac surgery at our centre between January 2018 and February 2024. Femoral access was achieved via open cut-down in 377 patients and percutaneous cannulation using a single ProGlide system in 376. The percutaneous cohort was categorized into 2 subgroups: early era (2020–2021, *N* = 102) versus late era (2022–2024, *N* = 274). The primary end-point was late access-site-related complications, while the secondary end-point was device failure requiring open femoral revision.

**RESULTS:**

Access-site complications including lymph fistula, healing disorders were significantly higher in the open cut-down group compared to the percutaneous group (lymph fistula: 10.6% cut-down vs 0.3% percutaneous, *P *=* *0.004, healing disorders: 3.4% cut-down vs 0% vascular closure device, *P *<* *0.001). The success rate of a single vascular closure device strategy improved from 65.7% in the early era to 91.6% in the late era (*P *<* *0.001), with fewer device failures (2.9% vs 11.7%; *P *=* *0.001).

**CONCLUSIONS:**

Percutaneous cannulation using single ProGlide is observed as a safe and feasible alternative to open femoral cut-down and showed excellent results after an initial learning curve.

## Graphical Abstract

**Figure:**
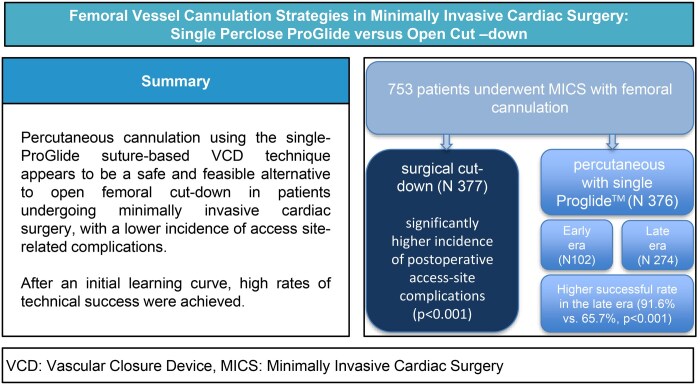

## INTRODUCTION

In recent years, there has been a growing trend towards minimally invasive cardiac surgery (MICS) of the atrioventricular valves, driven by both advancements in surgical techniques and patient preferences for less traumatic incisions. MICS offers several advantages over conventional cardiac surgery via a median sternotomy, including reduced postoperative pain, faster recovery and earlier resumption of physical activity [1]. A common technique in MICS is the femoral vessel cut-down and open vessel cannulation for initiation of cardiopulmonary bypass (CPB). However, open vessel cut-down is associated with wound healing complications at the groin site that may delay early patient recovery and hospital discharge and, thereby, minimize the clinical benefits of the MICS approach for the patient [2].

Vascular closure devices (VCDs) are widely used in transcatheter aortic valve implantation procedures and facilitate safe and effective percutaneous femoral vessel closure by providing excellent hemostasis [3]. The routine use of VCDs reduces wound healing complications when compared to open femoral vessel cut-down and accelerates patients’ ambulation after the procedure [4]. Various studies have consistently demonstrated that VCDs reduce the duration of surgery/intervention, length of stay in the intensive care unit (ICU) and hospital compared to open vessel cut-down [2, 5, 6].

The application of VCDs for achieving hemostasis following percutaneous femoral cannulation in MICS has gained increasing attention in recent years, especially, in high-volume transcatheter aortic valve replacement and MICS centres with large expertise in percutaneous femoral access techniques. In this study, we evaluated the safety and efficacy of using primarily a single suture-based VCD (Perclose ProGlide, Abbott, Chicago, IL) compared to the traditional open femoral vessel access in MICS. We also explored the feasibility of this single-device approach and analysed its associated learning curve.

## PATIENTS AND METHODS

A retrospective review of our institutional database was performed for all patients who underwent MICS between January 2018 and January 2024. A total of 753 patients (mean age: 63 years, male: *N* = 468, 62.2%) were included in this study. The study protocol was approved by the local ethical committee at Justus Liebig University in Giessen on 30 May 2023, with reference number AZ: 46/23. The collection and storage of data for our study follows the requirements outlined in the WMA Declaration of Taipei. The establishment and ongoing use of the databases have been approved and are monitored by our research ethics committee. Due to the retrospective nature of our study, written informed consent from the participants was waived. A detailed explanation of this waiver has been provided to and approved by the ethics committee.

Patients were stratified into 2 groups based on femoral vessel access methods: Conventional surgical femoral cut-down (cut-down group, *N* = 377) and percutaneous vascular cannulation (VCD group, *N* = 376) with femoral artery closure using single suture-based VCD (Perclose ProGlide, Abbott, Chicago, IL). The use of VCD was gradually adopted by 4 senior surgeons starting in 2020, and it became our standard cannulation approach for all MICS patients by the second half of 2021. To account for a possible learning curve, we categorized the VCD group patients into 2 subgroups: the early era of VCD use (2020–2021, *N* = 102) and the late era (2022–2024, *N* = 274). By this point, each surgeon had performed approximately 20–30 percutaneous cannulations and had developed the confidence and skill to safely perform the procedure and handle its complications.

The aim of our study was to retrospectively analyse the safety and efficacy of single ProGlide in MICS compared to open femoral cut-down. Demographic data, relevant pre- and intraoperative parameters and postoperative outcome data were obtained from our institutional patient records. The primary end-point of our analysis was late access-related vessel and wound complications, defined as femoral artery occlusion, lymph fistula, and wound infection at the access site. The secondary end-point was defined as device failure, requiring either conversion to cut-down by cannulation or open femoral artery revision by decannulation due to bleeding.

Until 2020, surgical cut-down with femoral arterial puncture under direct vision with purse-string closure remained the standard approach for the cannulation of femoral vessels in our centre. In 2020, ultrasound-guided percutaneous femoral cannulation with VCD was gradually introduced in MICS and later became the standard approach for groin vessel cannulation. The suitability of femoral vessels for cannulation was assessed through clinical examination and patient history. In general, a known peripheral vascular disease, previous femoral vessel surgery or arterial calcifications, as assessed by CT scan, were considered as contraindications for peripheral cannulation. In late 2023, a CT scan protocol (from the ascending aorta to the groin vessels) was implemented in all patients scheduled for MICS. The CT scan helped us plan our percutaneous cannulation strategy, including the cannulation side, cannulation level, and in very rare cases, the use of bilateral femoral cannulation in patients with small femoral arteries. If the CT scan showed severe atherosclerotic burden or peripheral artery disease in the descending aorta, the patient would be shifted to a sternotomy approach and automatically excluded from our study. In other words, the use of the CT scan has never led to shifting the patient from the VCD to the cut-down group but from femoral cannulation to other types of cannulation to reduce the complications of retrograde perfusion. To avoid bleeding complications, percutaneous access was exclusively achieved using ultrasound-guided infrainguinal transcutaneous needle puncture of the femoral vein and common femoral artery.

We typically use the left groin for arterial cannulation and the right groin for venous cannulation to simplify potential surgical revision in case of device failure. After a successful vessel puncture, guide wires are introduced into the descending aorta and superior caval vein under transoesophageal echocardiography (TEE) guidance, followed by heparinization. A single ProGlide is applied to the femoral artery over the guide wire at a 45° angle, followed by the insertion of the arterial cannula (17–19 F, HLS Cannulae, MAQUET/Getinge) using the Seldinger technique. The perfusion pressure is tested by the perfusionist, and the descending aorta is assessed by TEE before starting CPB. At the end of CPB and before decannulation, the arterial cannula is punctured and a guidewire is advanced into the descending aorta under TEE control. The arterial cannula is then removed, and the VCD knot is tightened according to the manufacturer’s instructions. If sufficient hemostasis is achieved, the guidewire is removed. If not, an additional ProGlide and/or AngioSeal VIP^®^ 6F (Terumo, Tokyo, Japan) can be applied using the existing guidewire. Protamine is administered to reverse heparinization. In cases of persistent bleeding, a surgical cut-down to the femoral artery was performed to control bleeding, either with direct stitches or vascular patch plasty. On the venous side, a horizontal mattress stitch was placed followed by manual compression for approximately 5 min. A pressure bandage was routinely applied to the arterial side for the first 6 h after surgery. Patients who were initially approached with percutaneous cannulation but had to be switched to surgical cut-down due to device failure, either during cannulation or decannulation, remained in the percutaneous group.

### Statistical analysis

Statistical analysis was performed using the IBM SPSS Statistics software package (Version 25; IBM Corporation, Armonk, New York, USA). Data are presented as mean ± standard deviation or as median with percentiles. The unpaired Student’s *t*-test or Mann–Whitney *U*-test was used for inter-group analysis, depending on the normality of data distribution. Normality of data distribution was assessed by histograms and the Shapiro–Wilk test. Categorical variables are expressed as counts and percentages and compared using Fisher’s exact test. To address selection bias and improve comparability between groups, we applied inverse probability weighting based on propensity scores to test our primary end-point. A *P*-value <0.05 was considered to indicate statistical significance.

## RESULTS

A total of 753 consecutive patients were included in this study. The demographic characteristics and baseline data of both groups are summarized in Table 1. There was no significant difference in gender distribution between the open cut-down group (*n* = 377, male 60.7%) and the VCD group (*n* = 376, male 63.5%, *P* = 0.452). The mean age of the cut-down group was 63.7 ± 12 years, compared to 62.9 ± 11.5 years in the VCD group (*P* = 0.380). Preoperative risk factors for wound healing disorders, such as body mass index, diabetes, and peripheral arterial disease were not significantly different between the groups (body mass index: 26.1 ± 4.6 vs 26 ± 4.3 kg/m^2^, *P* = 0.922; diabetes: 7.4% vs 8.8%, *P* = 0.506; peripheral arterial disease: 1.9% vs 2.4%, *P* = 0.623). However, the cut-down group had a higher incidence of previous stroke (6.9% vs 3.3%, *P* = 0.029), while poor mobility was similar in both groups (0.8% vs 0.3%, *P* = 0.319). Additionally, patients with pulmonary hypertension were more frequent in the cut-down group (49.2% vs 33.2%, *P* < 0.001), resulting in a higher EuroSCORE II in this group (1.5 ± 1.6 vs 1.2 ± 0.9, *P* = 0.003). There was no significant difference in STS-scores between the groups (1.3 ± 2.1 vs 1.3 ± 1.6, *P* = 0.765; Table 1).

**Table 1:** Pre- and intraoperative characteristics of MICS patients in the cut-down and VCD group

Parameter	Cut-down (*n* = 377) N (%) or mean ± SD	Proglide (*n* = 376) *N* (%) or mean ± SD	*P*-Value
Age	63.7 ± 12.0	62.9 ± 11.5	0.380
Sex, male	229 (60.7)	239 (63.5)	0.452
Body mass index (kg/m^2^)	26.1 ± 4.6	26 ± 4.3	0.922
Diabetes	28 (7.4)	33 (8.8)	0.506
COPD	17 (4.5)	20 (5.4)	0.617
Coronary artery disease	52 (13.8)	57 (15.3)	0.605
Peripheral vascular disease	7 (1.9)	9 (2.4)	0.623
Pulmonary hypertension	185 (49.2)	123 (33.2)	<0.001
Previous stroke	26 (6.9)	12 (3.3)	0.029
Poor mobility	1 (0.3)	3 (0.8)	0.319
Atrial fibrillation	137 (36.3)	119 (31.9)	0.294
Pre-cardiac surgery	8 (2.1)	8 (2.2)	1.000
Previous Mitral Clip	11(2.9)	5 (1.3)	0.113
STS-Score	1.3 ± 2.1	1.3 ± 1.6	0.765
EuroScore II	1.5 ± 1.6	1.2 ± 0.9	0.003
Mitral valve surgery	366 (97.1)	358 (95.2)	0.191
Repair	324 (88.5)	303 (84.6)	0.128
Replacement	42 (11.5)	55 (15.3)	0.128
Tricuspid valve surgery	27 (7.1)	34 (9.0)	0.419
ASD closure	8 (2.1)	9 (2.4)	0.812
Resection of myxoma	5 (1.3)	2 (0.5)	0.451
Cryoablation	86 (22.8)	93 (24.8)	0.549
Duration of surgery (min)	202 ± 56	229 ± 72	<0.001

Data are given as mean and standard deviation or median with interquartile range, and dichotomous data as count (%).

ASD: atrial septal defect; COPD: chronic obstructive pulmonary disease; VCD: vascular closure device.

The primary surgical procedure in both groups was either isolated or combined mitral valve surgery (cut-down: 97.1% vs VCD: 95.2%, *P* = 0.191), with comparable rates of mitral valve repair (88.5% vs 84.6%) and replacement (11.5% vs 15.3%, *P* = 0.128). Isolated or combined tricuspid valve surgery was performed at similar rates (cut-down: 7.1% vs VCD: 9.0%, *P* = 0.419). Additionally, the rate of atrial septal defect closure (cut-down: 2.1% vs VCD: 2.4%, *P* = 0.812) and myxoma resection (cut-down: 1.3% vs VCD: 0.5%, *P* = 0.451) were similar between groups (Table 1). The average duration of surgery was shorter in the cut-down group (202 ± 5.6 min) compared to the VCD group (229 ± 72 min, *P* < 0.001).

As depicted in Table 2, patients in the cut-down group had a significantly higher incidence of postoperative access-site complications compared to the VCD group (14.1% vs 0.5%, *P* < 0.001). Specifically, postoperative lymph fistula occurred in 10.6% of cut-down patients compared to 0.3% in the VCD group (*P *=* *0.004), and wound healing disorders requiring vacuum-assisted closure therapy were observed only in the cut-down group (3.4% vs 0%, *P* < 0.001), a difference that persisted even after risk-adjustment to account for possible selection bias (Table 2). Conversely, intraoperative bleeding requiring open femoral revision was more common in the VCD group (5.3%, *P* < 0.001).

**Table 2:** Outcomes and vascular complications in the cut-down and VCD group

	Cut-down (*n* = 377), Nr./mean (%)	Proglide (*n* = 376), Nr./mean (%)	*P*-value
Bleeding requiring open femoral revision*	0 (0)	20 (5.3)	<0.001
Late groin complications*	53 (14.1)	2 (0.5)	<0.001
Vascular occlusion	0 (0)	1 (0.3)	1.000
Lymph fistula	40 (10.6)	1 (0.3)	0.004
Healing disorder	13 (3.4)	0	<0.001
ICU stay (days)	2.4 ± 5.4	2.6 ± 3.8	0.053
Hospital stay (days)	12.1 ± 6.6	11.6 ± 6.4	0.378
Re-thoracotomy	9 (2.4)	8 (2.2)	1.000
ECMO support	5 (1.3)	6 (1.6)	0.771
Pacemaker	23 (6.1)	14 (3.8)	0.178
Dialysis	18 (4.8)	13 (3.6)	0.468
Stroke/TIA	5 (1.3)	9 (2.4)	0.297
In-hospital mortality	6 (1.6)	6 (1.6)	1.000

Data are given as mean and standard deviation or median with interquartile range, and dichotomous data as count (percentage).

* Risk-adjusted *P*-values using propensity score estimation and inverse probability weights.

ECMO: Extracorporeal membrane oxygenation; ICU: intensive care unit; TIA: transient ischaemic attack; VCD: vascular closure device.

Length of stay at ICU and index hospitalization was comparable between the 2 groups (ICU stay: cut-down 2.4 ± 5.4 vs VCD 2.6 ± 3.8 days, *P* = 0.053; hospital stay: cut-down 12.1 ± 6.6 vs VCD 11.6 ± 6.4 days, *P* = 0.378). In-hospital mortality was comparable in both groups (1.6%, *p* = 1.000). There were no significant differences in other postoperative events, including the rate of stroke/transient ischaemic attack, acute renal failure requiring hemofiltration or the need for extracorporeal membrane oxygenation (all *P* > 0.05) (Table 2).

To assess the learning curve of VCD within the MICS team, patients in the VCD group were divided according to the early (2020–2021) and late (2022–2023) adoption era (Table 3). Successful implementation of the single ProGlide strategy increased significantly in the late era (91.6% vs 65.7%, *P* < 0.001), thereby suggesting a steep learning curve. The need for additional VCDs to achieve sufficient hemostasis decreased also significantly in the late era (ProGlide: 34.3% vs 8.4%, *P* < 0.001; AngioSeal: 44.1% vs 28.8%, *P* = 0.007) with approximately two-third of all patients requiring no additional VCD for vessel hemostasis. Increased experience in the late era also led to significantly fewer device failures requiring open vessel revision (2.9% vs 11.7%, *P* = 0.002). Of note, only 1 patient in the early era experienced postoperative vascular occlusion and lymph fistula (Table 3).

**Table 3:** Access-site-related complications complication with respect to surgical era

	Era 2020–2021 (*n* = 102) *N* (%)	Era 2022–2023 (*n* = 274) *N* (%)	*P*-Value
Single Proglide^TM^	67 (65.7)	251 (91.6)	<0.001
Additional Proglide^TM^	35 (34.3)	23 (8.4)	<0.001
Additional AngioSeal	45 (44.1)	79 (28.8)	0.007
Device failure requiring open groin revision	12 (11.7)	8 (2.9)	0.002
Late groin complications	2 (2)	0 (0)	
Vascular occlusion	1	0	
Lymph fistula	1	0	
Healing disorder	0	0	
Hospital stay (days)	12.5 ± 7.5	11.3 ± 6	0.333

Data are given as mean and standard deviation or median with interquartile range, and dichotomous data as count (%).

## DISCUSSION

This retrospective study observed the safety and efficacy of percutaneous femoral cannulation using a single suture-based VCD (Perclose ProGlide™) for arterial closure in MICS. The use of VCD can eliminate access-related complications of the conventional open cut-down technique including lymph fistulas and wound infections. Nonetheless, acute vascular complications were observed more frequently in the VCD group due to device failure with persisting bleeding and femoral obstruction. Importantly, we clearly demonstrate that these VCD-related complications are significantly reduced after an initial learning curve and with increasing surgical expertise, although we cannot completely rule out that the late introduction of preoperative CT scans may have resulted in a selection bias with a less sicker patient population in the VCD group when compared to the surgical cut-down group.

VCDs are mainly used for transcatheter valve implantation and endovascular procedures. Therefore, most of the studies evaluating their safety and efficacy are from non-cardiac surgery patient populations. There is only few data on routine adoption of VCD for peripheral vessel cannulation in MICS patients. Previous studies reported overall complication rate after surgical cut-down for femoral vessel cannulation in MICS that range from 2.5% to 13.6% [2, 7, 8]. The wound complication rate in our study was 14.1% after femoral cut-down with the majority patients experiencing lymph fistulas (10.6%), a complication that occurs in 2–11% of patients after open femoral cannulation [2, 8, 9].

Another objective of our study was to evaluate the feasibility of a single Proglide VCD technique in MICS. Therefore, we did not use the Valve Academic Research Consortium (VARC)-2 or VARC-3 definitions of vascular complications, as they were developed for evaluating VCDs in the context of transcatheter valve interventions and do not include complications of conventional femoral cannulation techniques for MICS. Furthermore, major vascular complications such as aortic dissection or retroperitoneal bleeding are typically due to the percutaneous cannulation itself and not related to the application of the VCD. Surgical groin revision for relevant bleeding or vascular complications was necessary in 5.3% of all patients in the VCD group. Moschovas *et al.* reported a 2.3% revision rate with a 2 ProGlide approach in 353 patients undergoing MICS [2]. These low complication rates were also achieved after a growing expertise within our MICS team, as clearly shown by the significant reduction from 11.7% to 2.9% in our late VCD era. In another retrospective study of an alternate VCD device (Prostar, percutaneous arterial access suture-based closure device) used in 300 consecutive patients undergoing MICS with femoral vessel cannulation, bleeding complication rate was 4.3% and one patient (0.3%) experienced transitory claudication owing to superficial femoral artery thrombosis [10]. Only one patient (0.3%) in our VCD group experienced late vascular occlusion due to thrombosis, necessitating surgical revision. A vascular patch plasty was successfully performed, and the patient was discharged without any sequelae. This low incidence of femoral stenosis following the use of VCDs was also reported in other studies showing rates of 2.5% for the MANTA (Teleflex, Wayne, PA, USA) device and 0.8% for the 2 ProGlide devices [2, 7]. Compared to procedures performed in a hybrid room setting, we do not routinely control vessel patency or obstruction after VCD application via fluoroscopy in our MICS patients. However, we routinely perform a clinical assessment of distal leg perfusion in the operating room and at the ICU after removal of the femoral compression by Doppler examination.

The use of VCD and its impact on operation time and length of hospital stay remains controversial. Moschovas *et al.* reported a significant reduction in both duration of surgery and hospital stay. Similarly, El-Sayed Ahmad *et al.* found a reduction in the median procedural duration and ICU stay with VCD in 268 patients who underwent MICS using the plug-based MANTA. However, the length of hospital stay was not significantly different to the conventional cut-down group [2, 6]. We also failed to show a benefit of VCD use in MICS in terms of a reduction in the duration of surgery, length of stay at the ICU or hospital. In contrast, in our study, the duration of surgery in the VCD group was longer compared to the surgical cut-down group, a finding that can be attributed to the concomitant adoption of a total endoscopic 3D technique by the MICS team. Consequently, the duration of the overall procedure may have been biased by the change in the MICS approach overall.

After an initial learning curve of approximately 100 procedures performed by 4 surgeons over the first 2 years, the success rate of a single VCD strategy exceeded 90%, with a significant reduction in access-site complications and the need for an additional ProGlide or AngioSeal^®^. Conversely, Moschovas *et al.* reported no significant impact of the learning curve on the adoption of VCD, as there were no complications in the VCD group during the first 100 cases [2]. However, we believe that both percutaneous cannulation and implementation of VCD require a learning curve of at least 20–30 cases per surgeon. The idea behind using a single Proglide VCD, besides its cost-effectiveness, is that after tightening the VCD knot, the guidewire remains in place. This allows for the evaluation of the success of vascular closure and determines whether an additional AngioSeal or ProGlide is necessary to achieve sufficient hemostasis. Reifart *et al.* demonstrated in a study of 1105 patients who underwent transcatheter aortic valve replacement that the single ProGlide technique is feasible, with technical success rates comparable to those of the double-ProGlide technique [11].

Although our study did not aim to analyse the cost-effectiveness of a single VCD in MICS, we strongly believe from our experience that the reduction of vascular complications may lead to enhanced patient recovery, and this is clearly achieved by the use of VCD. A retrospective study of matched patients who underwent transcatheter aortic valve implantation, endovascular abdominal aortic aneurysm repair, thoracic endovascular aortic repair or balloon aortic valvuloplasty with arterial repair by either ProGlide or cut-down reported that the use of ProGlide was associated with significantly shorter index hospitalization and lower hospitalization costs compared with surgical cut-down [5]. Sekhar *et al.* have previously demonstrated the economic benefit of using the ProGlide^®^ VCD in the setting of percutaneous coronary intervention due to earlier discharge compared to conventional compression techniques [12].

### Limitations

Our trial has several limitations. No power analysis was performed due to the retrospective, monocentric design. However, our sample size appears to be justified when considering that in a post hoc sample size calculation, a total of 650 patients (325 per group, the incidence of the primary end-point 3% versus 8%, respectively) are needed to provide a power of 0.8 (type I error 0.05). Mid- or long-term clinical outcomes were not assessed in our study, thus, not allowing a valid interpretation of the long-term safety and efficacy of VCD in MICS. A potential selection bias or surgeon bias cannot be ruled out that may have influenced the inter-group differences with respect to access-site complications. On the other hand, all cannulation procedures, except for those performed on patients in 2020 and early 2021, were conducted with one strategy during each period. Other factors unrelated to femoral artery cannulation, such as the introduction of modern MICS techniques and changes in the surgical team during our study period may have led to a potential calendar time bias that may have influenced our primary and secondary outcomes. Importantly, the routine use of a preoperative CT scan was implemented in our institution in late 2023 (53 patients) and is a major limitation that may have influenced the results of our study. We strongly believe that CT-guided preoperative evaluation of the cannulation vessels is indispensable for a safe MICS procedure. We did not routinely perform a postoperative CT-angiography to account for possible femoral artery obstruction after VCD use, except for one patient with postoperative clinical signs of claudication.

## CONCLUSIONS

Compared to the surgical cut-down technique, the study suggested that the use of VCD is associated with a reduced rate of late access-site-related wound healing disorders. A single ProGlide technique for femoral arterial cannulation is safe and feasible and showed excellent results after an initial learning curve.

## Data Availability

The data underlying this article will be shared on reasonable request to the corresponding author.

## ABBREVIATIONS

CPB: Cardiopulmonary bypass
ICU: Intensive care unit
MICS: Minimally invasive cardiac surgery
TEE: Transesophageal echocardiography
VARC: Valve Academic Research Consortium
VCD: Vascular closure device
